# Integration of ATAC-Seq, Transcriptomic, and Proteomics Reveals the Molecular Mechanism of Intramuscular Fat Deposition and Meat Tenderness Regulation in Pig Breeds

**DOI:** 10.3390/biom15121738

**Published:** 2025-12-15

**Authors:** Yunpeng Zhang, Jing Xu, Suthar Teerath Kumar, Yunlong Zheng, Min Li, Ziyi Zhao, Qi Zhang, Wu-Sheng Sun, Li Pan, Yuan Zhao, Shu-Min Zhang

**Affiliations:** 1 Key Laboratory of Animal Production, Product Quality and Security, Ministry of Education, College of Animal Science and Technology, Jilin Agricultural University, Changchun 130118, China; zhangyunpeng@mails.jlau.edu.cn (Y.Z.); 20230033@mails.jlau.edu.cn (J.X.); kumar@mails.jlau.edu.cn (S.T.K.); zhengyunlong@mails.jlau.edu.cn (Y.Z.); limin56@mails.jlau.edu.cn (M.L.); 20230045@mails.jlau.edu.cn (Z.Z.); panli@jlau.edu.cn (L.P.); 2 Institute of Animal and Veterinary Sciences, Jilin Academy of Agricultural Sciences, Changchun 130033, China; zhangqi@mails.jlau.edu.cn; 3 College of Veterinary Medicine, Jilin Provincial Engineering Research Center of Animal Probiotics, Jilin Provincial Key Laboratory of Animal Microbiological Vaccine (Durg) for Major Animal Diseases, Ministry of Education, Jilin Agricultural University, Changchun 130118, China; sunwsh@jlau.edu.cn

**Keywords:** breeding, candidate genes, high-quality pork, IMF, multi-omics analyses

## Abstract

Pork is one of the most widely consumed meats worldwide, with tenderness and intramuscular fat (IMF) content serving as key determinants of consumer acceptance. The rising demand for high-quality pork underscores the need to better understand the molecular mechanisms regulating IMF deposition and meat tenderness. In this study, we systematically examined the tenderness and IMF in the *Longissimus dorsi* (LD) muscle of 104 eight-month-old Songliao black pigs and Leixiang pigs raised under identical conditions. In addition, three pigs from each breed were randomly selected for multi-omics analyses, including Assay for Transposase-Accessible Chromatin sequencing (ATAC-seq), transcriptomics, and proteomics to elucidate the molecular networks underlying IMF deposition and tenderness. We identified a total of 2635 differentially accessible chromatin (DARs) regions associated with 2006 functional genes and 351 regulatory transcription factors, predominantly enriched in adipocyte differentiation and muscle metabolism pathways. Transcriptome analysis revealed 624 differentially expressed genes (DEGs) involved in lipid metabolism and tissue structure maintenance. While proteomic profiling detected 153 differentially expressed proteins (DEPs) enriched in fatty acid degradation/metabolism, PPAR signaling, energy metabolism, and thermogenesis pathways. Further, combined integrated multi-omics analysis identified nine candidate genes (*MBP*, *DCLK1*, *COL3A1*, *ART3*, *COL14A1*, *PDK4*, *VCAN*, *LIPE*, *and GPX1*) and transcription factor–target interaction networks predicted key regulatory factors including MEF2A/C/D, PR, GR, AR-HALLSITE, NF1-HALLSITE, AP4, TCF21, MYOG, ATOH1, TCF12, BHLHA15, MYF5, ASCL1, and SIX2, which were potentially involved in the regulation of meat tenderness and IMF deposition. These findings provide novel insights into the molecular determinants of IMF and tenderness, offering valuable targets for improving meat quality through genetic breeding strategies.

## 1. Introduction

In recent years, with rapid socio-economic development in China, consumer demand for pork has shifted from quantity to quality [1]. Key quality indicators include pH, color, water loss rate, shear force, and intramuscular fat (IMF) content [2]. IMF is crucial because levels ≥ 2.5% substantially improve juiciness, tenderness, and flavor by promoting water retention and marbling within the muscle structure [3,4]. This is exemplified by native breeds such as the Lantang Pig, which shows superior meat quality linked to higher IMF and 22.3% lower shear force than Landrace [5,6]. Thus, the genetic improvement of IMF and shear force enables a direct translation of breeding progress into pork quality, offering a strategic solution to address the market’s demand for high-quality products at the source.

High-throughput Transposase-Accessible Chromatin sequencing (ATAC-seq) enables the identification of open chromatin regions where transcription factors bind to regulate gene expression, revealing epigenetic mechanisms underlying traits such as IMF [7,8,9]. RNA-seq enables the comprehensive identification of differentially expressed genes (DEGs) and the precise characterization of tissue-specific transcriptomic profiles under defined physiological conditions [10]. In addition, proteomics extends the analysis to the post-translational level, providing precise quantification of protein expression, modification status, and interaction networks [11]. Together, these technologies offer a key breakthrough for pig breeding by overcoming the limitations inherent in traditional selective breeding methods.

Due to the limited supply of high-quality pork products and increasing consumer demand, there is a potent concern about improving the swine meat industry through genetic improvement and breeding selection. The Songliao black pig is the first lean-type maternal breed in northern China. It has strong adaptability, a high reproductive rate, and fast growth and development, but the IMF content and tenderness need to be improved, which provides a high-quality lineage for the breeding characteristics of black pigs. Leixiang Pig is a local breed of wild boar, which is rich in IMF and delicate in meat quality [1]. The Songliao black pig and Leixiang Pig have a sharp contrast in meat quality traits, which is an ideal model for studying IMF deposition and meat tenderness. The research findings highlight that IMF and tenderness are very crucial indicators of meat quality, but the genes and regulatory molecular mechanisms controlling these traits are still unclear.

The Songliao black and Leixiang pig breeds display divergent meat quality phenotypes. This study aimed to elucidate the regulatory mechanisms of IMF deposition and meat tenderness in the *Longissimus dorsi* (LD) muscle through an integrated multi-omics approach, incorporating ATAC-seq, RNA-seq, and proteomic analyses. From the three dimensions of epigenetic regulation, gene transcription and protein expression, we constructed the regulatory network of “chromatin opening gene expression protein function” and identified several key transcription factors (TFs) and functional genes involved in the regulation of fat deposition and tenderness, which will provide an important theoretical basis for genetic improvement of pigs in future breeding strategies.

## 2. Materials and Methods

### 2.1. Ethical Statement

All experimental animals, from the Songliao black pig and Leixiang pig breeds, were raised at Feimasi Animal Husbandry Co., Ltd. (Changchun, China). Slaughtering and sampling were conducted under strict supervision in compliance with standard protocols. All animal care procedures were approved by the Animal Welfare Committee of Jilin Agricultural University (Approval No.: SYXK-2023-06-09-001).

### 2.2. Animal Housing, Feeding, and Sample Collection

In this study, a total of 104 pigs (52 Songliao black pigs and 52 Leixiang pigs) at 8 months of age during the fattening stage were selected as experimental subjects. The Songliao black pigs served as a control group, while the Leixiang pigs served as an experimental group. The nutritional value of the diet was in accordance with the national standard (NY/T 65-2021) [12], and the pigs were provided with ad libitum access to feed and water under the same conditions for 45 days. The humidity and temperature in the pigsty were maintained at 60–65% and 20–25 °C, respectively. At the age of 8 months, all pigs were slaughtered uniformly. A 20 g sample of the LD muscle was collected from each pig, quickly frozen in liquid nitrogen, and stored at −80 °C. Additionally, a 15 cm long fresh LD muscle tissue was taken for meat quality assessment.

### 2.3. Measurement of IMF and Shear Force

IMF and shear force measurement strictly followed the Chinese agricultural industry standard NY/T 821-2019 [13]. IMF content was quantified using the Soxhlet extraction method. Meat tenderness was assessed by measuring the peak shear force with a texture analyzer (Bulader, Beijing, China). From our initial population of 104 pigs, we ranked all individuals based on their IMF content and shear force values. Animals constituting the top 10% (highest IMF and lowest shear force) were assigned to the high-quality meat group, while those in the bottom 10% (lowest IMF and highest shear force) were assigned to the low-quality meat group. From the top and bottom 10% of samples ranked by IMF content and shear force, extreme phenotype groups were further selected for subsequent sequencing analysis: a high-IMF/low-shear group (n = 3) and a low-IMF/high-shear group (n = 3).

### 2.4. ATAC-Seq Analysis

In this study, ATAC-seq technology was employed to analyze chromatin accessibility in six samples (three for each breed). Paired-end sequencing (150 bp) was performed on the NovaSeq/DNBSEQ-T7 platform, with an average sequencing depth of 50 million reads per sample. Raw data quality control and filtering were conducted using FastQC and Fastp (v0.19.11). High-quality reads were aligned to the pig reference genome (*Sus-scrofa* 11.1) using Bowtie2 (v2.3.5.1). Open chromatin regions were identified using MACS2 (v2.1.2) (*p*-value < 1 × 10^−5^). Peak annotation was performed using ChIPseeker (v1.16.1). Differential accessibility regions were screened using DiffBind analysis (|log_2_Fold change (FC)| > 1, adjusted *p*-value (*p*) < 0.05) [9,10]. TF binding motifs within differentially accessible chromatin regions were predicted through de novo motif analysis using the MEME Suite (v5.4.1), with subsequent TF identification via TomTom database matching (E-value < 0.05). All data were visualized using IGV (v2.11.7).

### 2.5. RNA-Seq Analysis

Total RNA was extracted with TRIzol reagent, and samples with RNA integrity number (RIN) > 7.0 were used for cDNA library preparation. Sequencing was performed on the Illumina platform (paired-end, 150 bp). Raw reads were quality-checked with FastQC (v0.11.9) and filtered with Trimmomatic (v0.39). Clean reads were aligned to the reference genome using HISAT2, and gene expression was quantified by featureCounts and normalized as FPKM. To mitigate batch effects, samples were randomized during library preparation, and the median-of-ratios method from DESeq2 was applied for within-group normalization. Differential expression analysis was conducted using DESeq2 with thresholds (|log2FC| > 1 and adjusted *p* < 0.05) [10].

### 2.6. Four-Dimensional microDIA Groteomics Analysis

Total protein was extracted with SISPROT (Shenzhen Bepo Bio-Technology Co., Ltd., Shenzhen, China) lysis buffer, quantified by BCA assay (Beyotime Biotechnology, Shanghai, China), and 10 μg aliquots were digested with trypsin. Peptides were desalted using C18 cartridges, separated by nanoElute UHPLC on a C18 column with a formic acid/acetonitrile gradient, and analyzed on a timsTOF Pro 2 mass spectrometer in ddaPASEF mode (*m*/*z* 100–1700, 1/K0 0.7–1.4 Vs/cm^2^). Data were processed with DIA-NN (v1.8.1) using tryptic digestion (two missed cleavages permitted), carbamidomethylation (fixed), and oxidation/acetylation (variable), with FDR ≤ 1%. MaxLFQ was used for quantification, and differential expressions were assessed in Perseus (FC ≥ 1.5 or FC ≤ 0.6667, *p* < 0.05). Quality control included technical replicates and daily mass spectrometer calibration [14].

### 2.7. Functional Annotation of Expressed Genes and Proteins

Based on the Gene Ontology (GO) database (http://www.geneontology.org/ (accessed on 15 October 2024)), differential accessibility genes (DAGs), differentially expressed genes (DEGs), and differentially expressed proteins (DEPs) were functionally annotated, and their functional characteristics at three levels: Biological Process (BP), Molecular Function (MF), and Cellular Component (CC) were systematically analyzed. Pathway enrichment analysis was further performed through the Kyoto Gene and Genome Encyclopedia (KEGG) database (http://www.kegg.jp/ (accessed on15 October 2024)), and similar significantly enriched metabolic pathways and signal transduction pathways were identified for multi-omics analysis. Enrichment analysis was performed using Fisher’s exact test with FDR control via the Benjamini–Hochberg method (*p* < 0.05) [10,14,15].

### 2.8. Protein–Protein Interaction (PPI) and TFs-Target Gene Network

Through multi-omics integrative analysis, we identified common DEGs across different datasets. Based on transcriptomic and proteomic results, protein–protein interaction (PPI) networks were constructed using the STRING database (v12.0) and filtered for interactions with a medium confidence score ≥ 0.4 [16]. Based on ATAC-seq results, we further determined the TFs that bind to the regulatory regions of these common DEGs by analyzing the differential chromatin accessibility peaks [8]. The analysis results of the TF-target gene network construction were generated through the “MASS” package (v7.3-60) in R and visualized through CNSknowall (https://cnsknowall.com/ (accessed on15 April 2025)).

### 2.9. Real-Time Fluorescence Quantitative PCR

Total RNA was isolated from the LD muscle tissues of two groups of six pigs with TRIzol reagent (Thermo Fisher Scientific, Lenexa, KS, USA), followed by cDNA synthesis using SuperScript™ III First-Strand Synthesis SuperMix (Thermo Fisher Scientific, Lenexa, KS, USA) kit. To confirm the accuracy of RNA-Seq results, eight genes with significant expression differences were selected in this study for real-time fluorescence quantitative PCR verification. The primers used in the experiment were designed by Primer 6.0 software and synthesized by Shenggong Bioengineering Co., Ltd.(Shanghai, China) (Table S1). Primer amplification efficiency (95–105%) was validated by standard curve analysis, and reaction specificity was confirmed by the presence of a single peak in melt curve analysis. Quantitative PCR was performed on a CFX384 system (Bio-Rad Laboratories, Hercules, CA, USA) with three technical replicates per sample. Using β-actin as the internal reference gene, the Ct value method (2^−ΔΔCt^) was used to calculate the relative expression level of the target gene [16].

### 2.10. Statistical Analysis

SPSS 22.0 software was used for statistical analysis of the data. All data are presented as mean ± standard deviation (SD). Prior to analysis, all datasets were confirmed to satisfy the assumptions of normality (assessed by the Shapiro–Wilk test) and homogeneity of variances (assessed by Levene’s test). Based on this, inter-group comparisons were conducted using a two-tailed Student’s *t*-test. Differences were considered significant at *p* < 0.05 and highly significant at *p* < 0.01. It is noted that multiple testing correction was not applied to these specific comparisons as they were pre-planned and limited in scope.

## 3. Results

### 3.1. IMF and Shear Force Analysis

**Figure 1:**
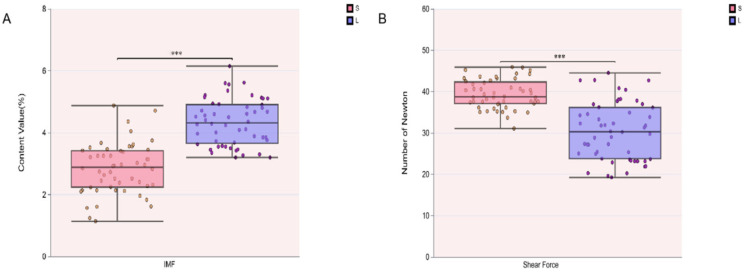
Meat quality trait analysis in Songliao black pig (S) and Leixiang pig (L) (n = 104). (**A**) IMF analysis. (**B**) Shear force analysis.*** represents *p* < 0.001.

The analysis of meat quality characteristics revealed that the LD muscle of Songliao black pigs had an IMF content of 2.84 ± 0.82% and a shear force value of 39.39 ± 3.46 N. In contrast, Leixiang pigs exhibited an IMF content of 4.33 ± 0.75% and a shear force of 30.43 ± 6.97 N in the same muscle (Table S2). Significant differences were observed in both IMF content and shear force between the two breeds (*p* < 0.05) (Figure 1A,B). Specifically, the Leixiang pig group showed a significantly higher IMF content (*p* < 0.05) and a significantly lower shear force (*p* < 0.05) compared to the Songliao black pigs. Results indicate that the overall model is fit for downstream analysis.

### 3.2. ATAC-Seq Quality Control

**Figure 2:**
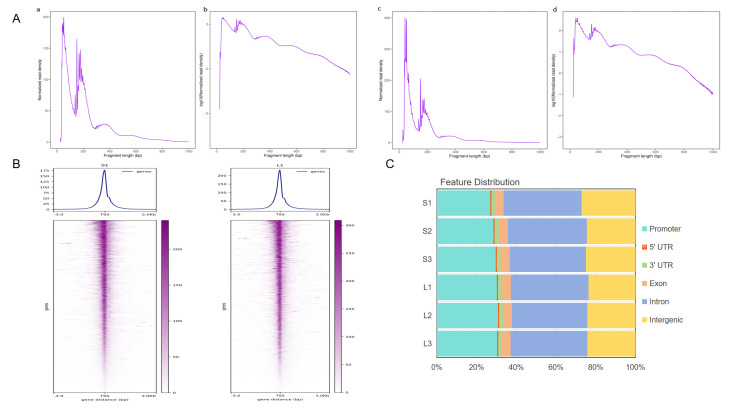

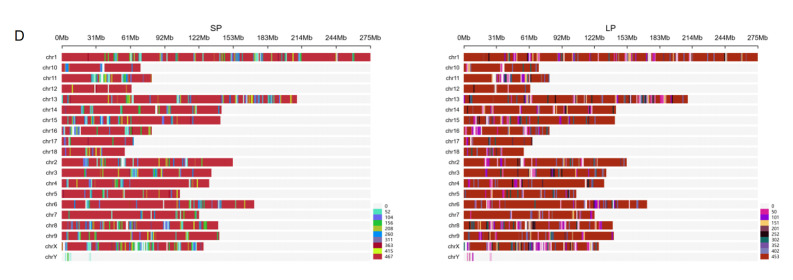
Peak distribution analysis. (**A**) Fragment length distribution map. (**B**) ATAC-seq signal enrichment in 3 kb regions upstream and downstream of TSS. (**C**) Distribution of peak sites in different genomic regions. (**D**) Distribution of peak sites in chromosomes.

ATAC-sequencing data showed that 510,596,262 and 541,780,720 original reads were obtained in group Songliao black pig and group Leixiang pig, respectively. After the filtration, 510,584,228 and 541,766,676 were retained, respectively, and the ratio of each sample exceeded 97% (reference genome: Sus scrofa 11.1) (Table S3). The analysis results of insertion fragment length in the library were consistent with the expected distribution. The leftmost peak represents the nucleosome-free region in open chromatin, and the characteristic peaks at 200 bp and 400 bp correspond to open chromatin in mononucleosome and binucleosome regions, respectively (Figure 2A). The sequencing reads were significantly enriched within the 3 kb range around the transcription start site (TSS), and obvious signal peaks were present near the TSS, indicating reliable data quality, as exemplified by samples S1 and L1 (Figure 2B). Peak analysis identified 104,363 and 94,787 open chromatin regions in the Songliao black pig and Leixiang pig groups, respectively, and genomic annotation indicated that these regions were mainly distributed in promoters, introns, and distal intergenic regions (Figure 2C). Genome-wide signal distribution analysis showed that the chromosomal open patterns of were highly consistent between the two groups of samples (Figure 2D).

### 3.3. Differences in Accessibility and Motif Analysis

**Figure 3:**
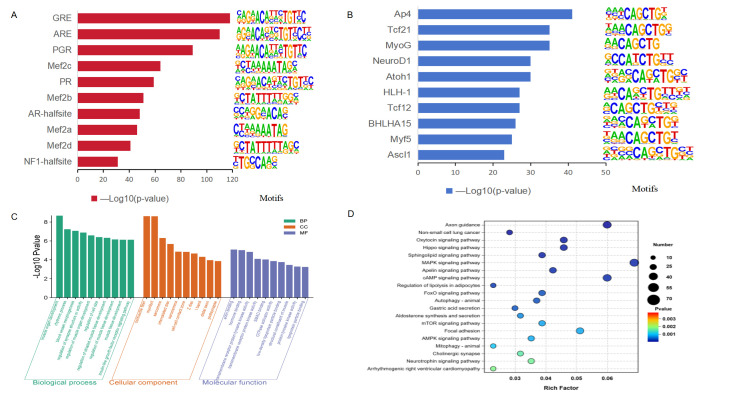
Transcription factor binding motif and enrichment analysis. (**A**) Transcription factor binding motif enriched by up-regulating peak sites. (**B**) Transcription factor binding motif enriched by down-regulating peak sites. (**C**) GO functional enrichment analysis of genes corresponding to different peaks. (**D**) KEGG pathway enrichment analysis of genes corresponding to different peaks.

Comparison of chromatin accessibility between Songliao black pig and Leixiang pig breeds identified a total of 2635 differential peaks by applying the screening criteria (|log2FC| > 1 and adjusted *p* < 0.05), which included 1568 up-regulated and 1067 down-regulated peaks. These regions were annotated, leading to the identification of 2006 differentially accessible genes (DAGs) (Table S4). Motif analysis found a total of 351 extremely significantly enriched transcription factor binding motifs in the differential open chromatin region (OCR) (*p* < 0.01) (Table S5). Top 10 motifs with the highest enrichment in the up-regulated OCR region mainly include adipogenesis-related transcription factors such as GR, AR, PR/PGR, and Mef2 families (Figure 3A); while the down-regulated OCR region significantly enriches myocyte differentiation-related transcription factors such as Ap4, Tcf21r, MyoG, and NeuroD1 (Figure 3B).

### 3.4. Functional and Cluster Annotations of DAGs

By integrating GO functional annotation and KEGG pathway enrichment analysis, the biological functions of DAGs in IMF deposition and tenderness regulation were systematically analyzed. GO analysis showed that DAGs were significantly enriched in three key functional modules (Figure 3C,D, Table S6). In terms of biological processes, enrichment was mainly observed in muscle organ development, rhythm regulation, and vascular morphogenesis. Regarding cell components, it focuses on muscle-specific structures such as contractile fibers, myofibrils, and sarcomeres. For molecular functions, it is significantly enriched in actin binding, hormone binding, and transmembrane receptor protein tyrosine kinase activity. KEGG pathway analysis identified 90 significantly enriched metabolic pathways (Table S7), of which the top 20 pathways were mainly involved in fat metabolism and myocyte energy regulation, including the MAPK signaling pathway, Apelin signaling pathway, cAMP signaling pathway, fat decomposition regulation, FoxO signaling pathway, mTOR signaling pathway, AMPK signaling pathway, mitochondrial autophagy, and focal adhesion, among others.

### 3.5. RNA-Seq Quality Control

A total of 334.68 million clean reads were obtained in transcriptome sequencing analysis, with an overall comparison rate of 98.20%. The data quality of each sample is excellent, the number of clean reads is 41.41–67.63 million, the comparison rate is 97.79–98.48%, and the proportion of Q30 bases is 94.34–96.90% (Table S8). Based on quantitative analysis of the FPKM algorithm, a total of 19,156 expressed genes were detected in this study, of which 16,009 genes were stably expressed in all samples.

### 3.6. Comparison Analysis of RNA-Seq and Functional Annotations

**Figure 4:**
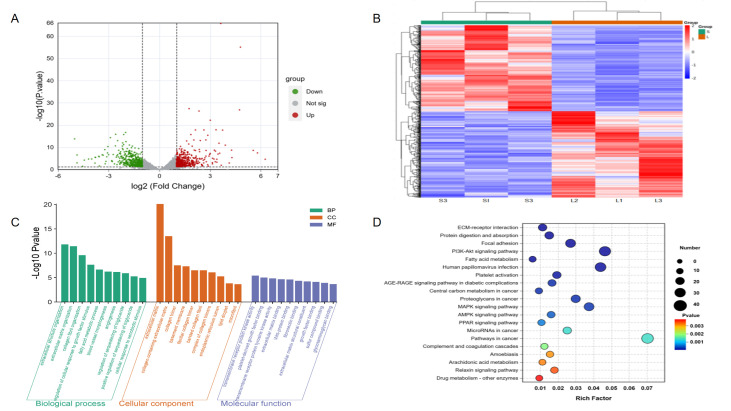
RNA-seq data analysis. (**A**) DEGs volcanic map. (**B**) DEGs heat map. (**C**) DEGs GO functional enrichment analysis. (**D**) DEGs KEGG pathway enrichment analysis.

We identified a total of 624 DEGs based on (|log_2_FC| ≥ 1 and adjusted *p* < 0.05) between Songliao black pig and Leixiang pig breeds. Among them, the Leixiang pig breed showed 331 up-regulated genes and 311 down-regulated genes compared with the Songliao Black pig group (Figure 4A,B). The biological functions of 624 DEGs in IMF deposition and tenderness regulation were analyzed by the GO function annotation (Figure 4C, Table S9). The results showed that DEGs are mainly involved in three key functional modules. In terms of biological processes, they are significantly enriched in extracellular structural tissue, extracellular matrix tissue, and collagen fiber formation; in terms of cell components, they are mainly located in extracellular matrix, collagen trimer, and other structures; and in terms of molecular functions, they are significantly enriched in signal transduction-related functions such as transmembrane receptor protein kinase activity and platelet-derived growth factor binding. KEGG pathway enrichment analysis revealed the key regulatory role of differentially expressed genes (DEGs) in fat metabolism and tissue structure maintenance (Figure 4D, Table S10). DEGs involve 299 KEGG pathways. Among them, 52 were significantly enriched pathways. The first 20 pathways are mainly divided into two major functional modules: lipid metabolism-related pathways, including PPAR signaling pathways and fatty acid metabolism; and tissue structure-related pathways, such as extracellular matrix (ECM) receptor interaction and focal adhesion. It is worth noting that ECM-receptor interaction, protein digestion and absorption, and focal adhesion are the three most abundant pathways.

### 3.7. Four-Dimensional microDIA Quantitative Proteomics Quality Control

Data independent acquisition (DIA) mass spectrometry analysis identified 3430 proteins corresponding to 32,104 unique peptides of Songliao black pig, and 31,550 peptides of Leixiang pig. Quality control analysis showed the length distribution of the peptides was mainly concentrated in the 7–20 amino acid interval, which meets the characteristics of trypsin enzymatic lysis. Protein credibility was positively correlated with coverage, with high-confidence proteins (including polypeptides) predominating. The enzymatic lysis efficiency was excellent, and the proportion of peptides in single-enzyme cleavage sites reached 78.75%, indicating that the sample pretreatment quality is reliable.

### 3.8. Functions and Cluster Annotations of DEPs

**Figure 5:**
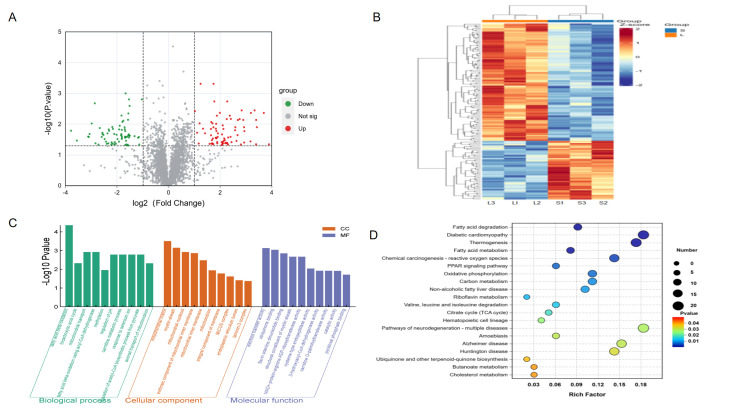
Proteomics data analysis. (**A**) DEP volcanic map. (**B**) DEP heat map. (**C**) DEP GO functional enrichment analysis. (**D**) DEP KEGG pathway enrichment analysis.

We identified 153 differentially expressed proteins (DEPs) between the Leixiang pig and the Songliao black pig breeds (FC ≥ 1.5 or FC ≤ 0.6667, *p* ≤ 0.05). Among them, 102 proteins were up-regulated in the Songliao black pig breed, and 51 were down-regulated in the Leixiang pig breed (Figure 5A,B). GO functional annotation showed that DEPs were mainly involved in three core functional modules (Figure 5C, Table S11), biological processes, and metabolic processes such as β-oxidation, tricarboxylic acid circulation, and mitochondrial transport. Cellular components are mainly located in key places for energy metabolism, such as the mitochondria and the mitochondrial matrix. Molecular functions are prominently manifested in electron transfer-related functions such as flavin adenine dinucleotide binding and oxidoreductase activity. KEGG pathway analysis showed that the DEPs were involved in 218 pathways, and 22 significantly enriched pathways were identified (Figure 5D, Table S12), which were mostly related to fat formation and metabolism, such as fatty acid degradation, fatty acid metabolism, PPAR signaling pathways, and thermal production. These pathways were also significantly enriched in the transcriptome data, indicating that lipid metabolism-related pathways play an important regulatory role in transcriptional and translational levels in IMF deposition.

### 3.9. Multi-Omics Correlation Analysis of ATAC-Seq, RNA-Seq, and Proteome Sequencing

**Figure 6:**
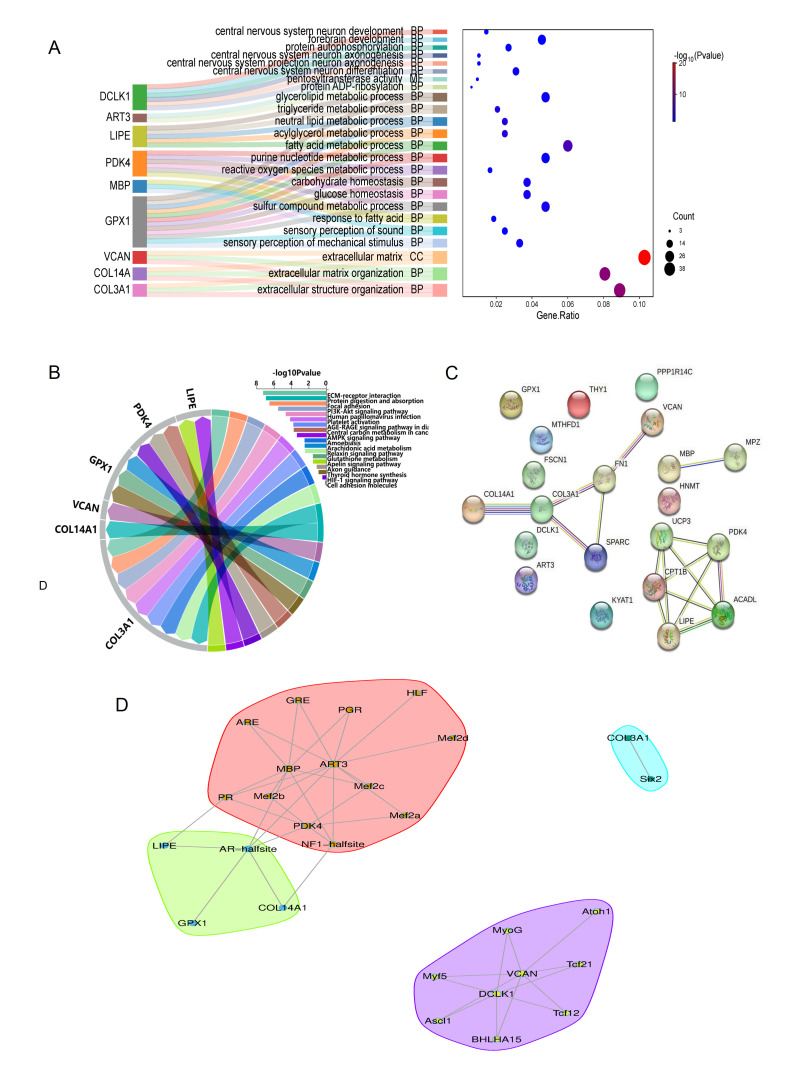
Integrated data analysis. (**A**) Functional enrichment analysis of candidate genes GO. (**B**) KEGG pathway enrichment analysis. (**C**) Protein–protein interaction analysis. (**D**) TF-target gene interaction analysis.

By integrating RNA-seq and proteomic data, 24 candidate genes related to lipid deposition and tenderness of meat, such as *MBP*, *DCLK1*, and *COL3A1,* were initially screened. Furthermore, integration with ATAC-seq data identified 9 key regulatory genes, *PDK4*, *LIPE*, *GPX1*, *ART3*, *MBP*, *COL3A1*, *COL14A1*, *VCAN,* and *DCLK1*. Functional enrichment analysis of GO and KEGG showed that extracellular matrix regulation was significant (Figure 6A,B), with *COL3A1*, *COL14A1,* and *VCAN* markedly enriched in extracellular matrix tissues (GO: 0043062). *COL3A1* was enriched in the ECM-receptor interaction pathway (ko04512), and *VCAN* was significantly enriched in the cell adhesion molecular pathway (ko04514), suggesting that it may affect meat tenderness by regulating muscle tissue structure. Lipid metabolism regulation: *PDK4* and LIPE participate in energy metabolism through HIF-1 (ko04066) and AMPK signaling pathways (ko04152), respectively, while *GPX1* regulates glutathione metabolism (ko00480). The three together form a fat metabolism regulatory network. Neuro-related functions: *MBP* is mainly involved in myelination (GO: 0042552), and *DCLK1* regulates neuronal differentiation (GO: 0021953); although its precise function has not yet been fully clarified. *ART3* mainly acts on post-translational modification of proteins through two processes: ADP-ribosylation (GO: 0006471) and pentylglycosyltransferase activity (GO:0016765). The functional mechanism of *ART3* still needs further exploration. It is worth noting that the synergistic effects of *PDK4-MBP* and *LIPE-GPX1* may play a key role in fat metabolism, and these findings reveal the central role of extracellular matrix remodeling and energy metabolism regulation in determining flesh tenderness and fat deposition.

### 3.10. Protein–Protein Interaction and TFs-Target Gene Interaction Network

To reveal the protein interaction network during fat deposition in the LD muscles of Leixiang pigs, a protein interaction network was constructed using DEGs and DEPs shared by the transcriptome and proteome (Figure 6C). There are various forms of protein–protein interactions, and 12 interacting proteins related to key pathways were detected: *COL3A1*, *COL14A1*, *FN1*, *SPARC*, *VCAN*; *PDK4*, *UCP3*, *LIPE*, *ACADL*, *CPT1B*, *MBP*, and *MPZ*. These proteins are involved in different pathways related to fat deposition and tenderness regulation.

Through an integrated analysis of ATAC-seq peaks associated with co-differentially expressed genes across the three omics layers—where associated genes were defined as the nearest genes in physical proximity to the peaks and considered as potential regulatory targets—notable patterns emerged. We found that target genes of different functional categories had characteristic transcription factor regulation patterns (Figure 6D). The promoter regions of neurodevelopment-related genes *DCLK1* and *VCAN* are rich in a variety of myogenic transcription factor binding sites, including bHLH family members (ASCL1, ATOH1, and BHLHA15), myocyte determinants (MYOG, MYF5), and regulatory elements such as AP4, TCF21, and TCF12. The key genes of energy metabolism, *PDK4, ART3,* and *MBP* are mainly affected by nuclear receptor families (PGR, PR, and AR-halfsite), the MEF2 family (MEF2A/B/C), and the NF1-halfsite. The extracellular matrix genes *COL3A1* and *COL14A1* are specifically regulated by SIX2 and the AR-halfsite/NF1-halfsite elements, respectively. The core regulatory factors of lipid metabolism, *LIPE* and *GPX1,* both contain AR-halfsite elements, among which *LIPE* is also regulated by PR. In the Leixiang pigs with high intramuscular fat (IMF_H), chromatin accessibility, transcriptional levels, and protein abundance of *GPX1*, *LIPE*, *PDK4*, *MBP,* and *ART3* were consistently higher compared to those in the Songliao black pigs with low intramuscular fat (IMF_L). Conversely, *DCLK1*, *VCAN,* and *COL3A1* exhibited lower chromatin accessibility, mRNA expression, and protein levels in Leixiang pigs relative to the IMF_H group. Intriguingly, although *COL14A1* demonstrated higher chromatin accessibility in Leixiang pigs, both its transcript and protein levels were reduced compared to those in Songliao black pigs.

### 3.11. Validation of RNA-Seq Results Using qRT-PCR

**Figure 7:**
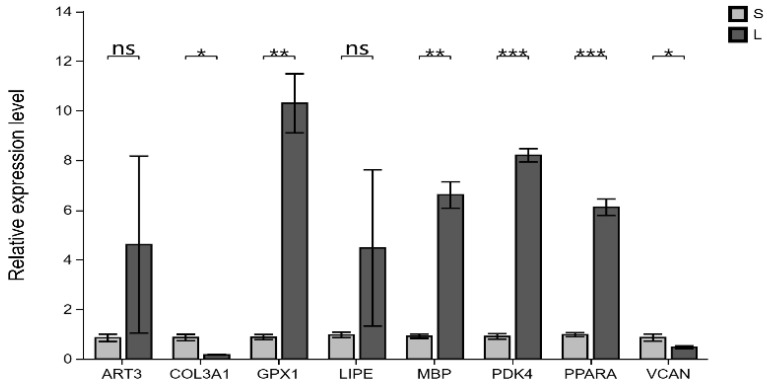
Validation of RNA-seq data by RT-qPCR. Relative expression levels measured by the y-axis RT-qPCR. Mean ± standard deviation, * represents *p* < 0.05; ** represents *p* < 0.01; *** represents *p* < 0.001; ns represents *p* > 0.05.

The qRT-PCR test results showed that the expression trends of eight genes in the LD muscle samples were consistent with the transcriptome analysis results (Figure 7). This indicates that transcriptome sequencing results are reliable.

## 4. Discussion

Intramuscular fat content and tenderness are key factors that determine pork quality, and their formation is regulated by a dynamic balance of fat generation and decomposition. In this study, we used Songliao black pigs and Leixiang pigs with significant differences in IMF and shear force as models. By integrating ATAC-seq, Transcriptome, and Proteome analysis, we successfully identified key genes and transcription factors related to differences in IMF and shear force.

### 4.1. Network of Key Transcription Factors in Differentially Open Chromatin Regions

This study revealed a network of key transcription factors that regulate pork quality through chromatin accessibility analysis. In the OCR up-regulated by the Leixiang pig group, transcription factor binding sites such as GRE, ARE, PRE, and MEF2 were significantly enriched, while the down-regulated region was mainly enriched for binding sites such as AP4, TCF21, and MYOG. Only the top 10 key transcription factors for up- and down-regulation were screened. Notably, all the down-regulated factors contained the bHLH domain, suggesting that this domain plays an important role in the regulation of muscle development and fat deposition.

In up-regulated OCRs, MEF2 family transcription factors participate in muscle development and fat metabolism processes by regulating target genes such as *ART3*, *MBP,* and *PDK4*. Among them, MEF2A/C/D is crucial for muscle regeneration and differentiation [17], while MEF2C/D can also improve tenderness of flesh by promoting slow muscle fiber differentiation [18]. In addition, MEF2 family members are also involved in fat metabolism regulation, such as affecting fatty acid oxidation and brown fat thermal production [19]. The study also identified important nuclear receptors such as the Progesterone receptor (PR) and Glucocorticoid receptor (GR). The Progesterone receptor has binding sites in genes such as *ART3*, *MBP*, *PDK4,* and *LIPE*. The target genes of the transcription factor GR are *ART3* and *MBP*. They regulate lipid metabolism-related gene expression by binding to corresponding reaction elements [20,21]. Of particular note, AR-halfsite and NF1-halfsite are involved in skeletal development and extracellular matrix remodeling by targeting *COL14A1* [22,23].

In the down-regulation of OCRs, we identified myofibers such as AP4, TCF21, MYOG, ATOH1, TCF12, BHLHA15, MYF5, and ASCL1, and their target genes are mainly *DCLK1* and *VCAN*. These factors affect flesh quality characteristics through different mechanisms, and MYOG regulates myofibers and contractile protein expression [24]; MYF5 affects myofibers through IGF-1 signaling [25]; and TCF21 is involved in myofibers and collagen synthesis regulation [26]. In addition, AP4 participates in succulent formation by influencing extracellular matrix remodeling [27], while SIX2 participates in the tissue repair process by targeting *COL3A1*. These findings construct a network of transcription factors for pork quality regulation, providing a theoretical basis for the genetic improvement of meat traits.

### 4.2. Analysis of Common Differential Functional Genes and Proteins

By performing GO function and KEGG pathway enrichment analysis on differential genes, genes related to lipid deposition and tenderness were screened, and a total of nine significantly differentially expressed genes with consistent expression trends were identified. Compared with Songliao black pigs, the expression of *MBP*, *ART3*, *PDK4*, *LIPE*, and *GPX1* genes in the LD of Leixiang pigs was significantly up-regulated, while the expression of *DCLK1*, *COL3A1*, *COL14A1*, and *VCAN* genes was significantly down-regulated. It is speculated that the above genes are candidate genes that affect the deposition and tenderness of pigs.

This study systematically reveals the regulatory role of multiple key genes in pig fat metabolism and meat formation. *PDK4*, as a core regulator of energy metabolism, is highly expressed in skeletal muscle and adipose tissue [28], and its activity is regulated by trophic status and motor [29]. *PDK4* promotes fatty acid oxidation and maintains glucose homeostasis by inhibiting the pyruvate dehydrogenase complex [30,31]. This study shows that *PDK4* is significantly enriched in biological processes such as blood sugar homeostasis, carbohydrate homeostasis, and reactive oxygen metabolism. In a number of comparative studies on pig breeds, the expression level of *PDK4* is significantly correlated with the fat deposition characteristics of different pig breeds, such as the Taoyuan black pig and Duroc pig [32], Nanyang black pig [16], Berkshire and Jeju pig [15], Wei and Yorkshire pig [33], and the Belgian commercial hybrid pig [34]. Genetic analysis shows that the *PDK4* gene polymorphism directly affects meaty traits such as IMF content and backfat thickness [35,36]. *PDK4* knockout mice demonstrated that its deletion can lead to a decrease in blood sugar after fasting, a decrease in fatty acid oxidation rate, and an increase in glucose and pyruvate oxidation [37]. *LIPE*, as a key rate-limiting enzyme in the lipolysis process, affects glucose uptake and insulin sensitivity in muscle tissue through glucose homeostasis signaling pathways [38]. This gene significantly affects IMF deposition by regulating free fatty acid mobilization and lipid homeostasis [39]. *LIPE* plays a dual regulatory role in fat metabolism. Developmental dynamic analysis shows that *LIPE* expression patterns are closely related to the fat deposition process. Upregulation in the late development of Tibetan pigs in Diqing promotes IMF accumulation [40], while in the early development stage of Laiwu pigs, it coordinates with *GPAT4* to regulate lipid metabolism. Lipid absorption may be the main reason for the increase in adipogenesis [41]. In this study, fat digestion and absorption pathways were significantly enriched. Comparative transcriptome studies revealed that the *LIPE* expression level in fat-type pig breeds is significantly higher than that in lean-type pig breeds [32]. However, its regulatory model is breed- and tissue-specific. Its high expression in cattle adipose tissue significantly inhibits the expression of cellular adipogenesis genes and promotes fat metabolism [42]. In the semimembrane muscles of large white pigs in Italy, samples with lower IMF content showed higher *LIPE* levels [43]. Genetic marker analysis found that the *LIPE* gene polymorphism not only affects the IMF content but also significantly correlates with fatty acid composition [39]. It is worth noting that *LIPE* and *PDK4* may have functional complementarity in fat metabolism. *PDK4* promotes fatty acid utilization, while *LIPE* regulates fat mobilization. Antioxidant systems also play an important role in the formation of flesh. *GPX1* protects cellular function by maintaining redox balance, and its expression level is closely related to IMF content and fatty acid composition [44,45]. This study found that *PDK4* and *GPX1* were significantly expressed in high IMF tissues, and the two were involved in the processes of sulfur compound metabolism, blood sugar homeostasis, and reactive oxygen metabolism. *LIPE* and *GPX1* are enriched in processes such as fatty acid and acylglycerol metabolism. This shows that *GPX1* has a collaborative expression pattern with *PDK4* and *LIPE*, forming a “metabolic-antioxidation” regulatory network. *PDK4* and *LIPE* regulate energy metabolic flow, while *GPX1* maintains metabolic enzyme activity by removing reactive oxygen species [46]. This fine regulatory mechanism may be one of the key factors that determine the quality of pork. In addition, the study also found that *MBP*, a neurospecific protein, is abnormally expressed in high-fat tissues [47,48]. Although its specific mechanism of action is not yet clear, the co-localization of *MBP* and *PDK4* in the Fatty acid response pathway suggests that nervous system-related proteins may participate in peripheral fat metabolism regulation through unknown pathways. These findings provide new research directions for a deep understanding of the molecular mechanisms of pork quality formation.

Unique regulatory roles of *DCLK1* and *ART3* in pig muscle development and fat metabolism. As a multifunctional protein kinase, *DCLK1* not only participates in neuronal differentiation by regulating microtubule dynamics [49], but also plays an important role in metabolic disorders. In tumor biology, it is involved in the development of multiple malignant tumors as a pro-cancer factor [50]; in obesity models, *DCLK1* promotes myocardial fibrosis by activating the RIP2/TAK1 pathway [51], suggesting that it may affect fat metabolism by regulating the inflammatory microenvironment. It is worth noting that *DCLK1* may also indirectly affect flesh tenderness by regulating cytoskeleton dynamics and muscle protein degradation [52], which provides a new perspective for understanding muscle quality formation. As a metabolic regulator, *ART3* is specifically highly expressed in skeletal muscle, and its expression level is negatively correlated with muscle fat infiltration [53,54]. During the development of flower pigs in southern Anhui, *ART3* was involved in the regulation of muscle fat deposition [14]. The upregulation of *ART3* expression in high-fat pigs in this study further confirmed this association. Mechanism studies show that *ART3* regulates protein function and signal transduction through ADP-ribosylation modification [55], but its precise pathway of action in fat metabolism still needs to be further analyzed. In particular, the versatility of *DCLK1* and *ART3* in different physiological processes suggests that they may play a central role in connecting muscle development, fat metabolism, and fleshy properties.

Extracellular matrix (ECM)-related genes play a key regulatory role in flesh formation. COL3A1 (type III collagen), COL14A1 (type XIV collagen), and VCAN (glass-like protein) are core components of the ECM, and regulate tissue structure and function by constructing complex protein interaction networks [56,57,58]. Collagen is a major component of the ECM, which determines the structural support and strength of the ECM in connective tissues [59]. Studies have shown that collagen content is closely related to fleshy properties, positively correlated with shear force values, and negatively correlated with tenderness [60]. *COL3A1* expression is negatively correlated with collagen solubility, and increasing collagen solubility improves tenderness [61,62]. Fat deposition can improve fleshy quality by changing the ECM structure, such as fat infiltration destroying connective tissue [63]. It is worth noting that these ECM genes are also involved in fat metabolism regulation. *COL14A1* is down-regulated in Iberian pigs with excellent tenderness [64], Southern Anhui Flower pigs [65] and Wei pigs [33], and is also involved in preadipocyte differentiation and regulation of backfat thickness [60,66,67]. *VCAN* participates in metabolic regulation by affecting adipose tissue growth and angiogenesis, and affects changes in fatty acid content and glycogen content in muscles [68,69], and down-regulates expression in fatty pig breeds [70,71]. FN1 (fibronectin) is a key ECM protein [72] and is significantly differentially expressed in the transcriptome and proteome. VCAN forms a complex protein–protein interaction regulatory network through the indirect interaction between FN1 and COL3A1, which jointly affects the structure and function of the extracellular matrix. This study found that COL3A1, COL14A1, VCAN, and FN1 were synergistically down-regulated in high-IMF and high-tenderness Thunder pigs, suggesting that ECM reconstruction plays an important role in meat tenderness and fat deposition.

### 4.3. Combined Analysis Results

**Figure 8:**
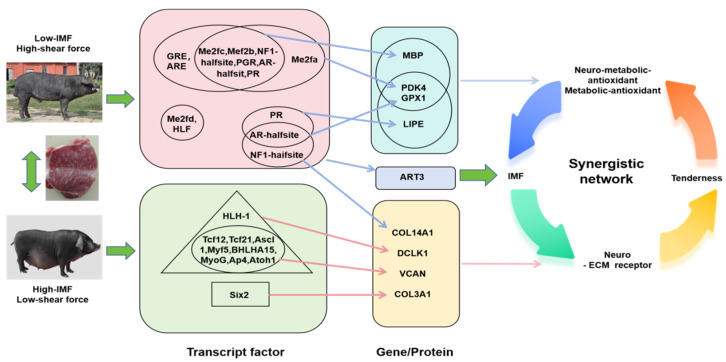
Visualization of the integrated multi-omics analysis findings of transcription factor, gene, and protein regulation, illustrating the molecular mechanism of two different pig breeds in shear force and intramuscular fat (IMF) in the LD muscle.

This study systematically analyzes the molecular mechanism of pork quality formation through integrating chromatin accessibility analysis and transcriptional regulation, and protein interaction network. Analysis of transcription factor-target gene regulation network shows that *DCLK1* and *VCAN* are regulated by muscle fiber type transcription factors such as AP4, TCF21, and MYOG; *PDK4*, *ART3,* and *MBP* are regulated by MEF2 family and nuclear receptors (PR, GR, etc.); *LIPE* and *GPX1* are regulated by transcription factors such as AR-halfsite; and *COL3A1* and *COL14A1* are regulated by SIX2 and AR-halfsite/NF1-halfsite, respectively. Analysis of key regulatory gene function. In terms of energy metabolism, *PDK4* promotes fatty acid oxidation by regulating sugar metabolism, and *LIPE* affects lipid metabolism by regulating lipolysis. The two jointly maintain the balance of intramuscular fat deposition. In terms of antioxidants, *GPX1*, *PDK4*, and *LIPE* form a “metabolic-antioxidation” collaborative network. In terms of extracellular matrix, *COL3A1*, *COL14A1,* and *VCAN* jointly regulate the tenderness of flesh by constructing ECM structures, among which *COL14A1* and *VCAN* may also participate in the IMF deposition process. Newly discovered regulatory factors, *MBP,* may indirectly affect fat deposition through neuro-metabolic coupling; *ART3* participates in fat metabolism regulation through signal transduction, and *DCLK1* functional pleiotropy suggests its potential regulatory effect. These findings not only reveal the multi-level regulatory network formed by pork quality, including key processes such as transcriptional regulation, energy metabolism, oxidative balance, and extracellular matrix remodeling, but also provide new targets and a theoretical basis for pig molecular breeding. In particular, the discovered “metabolic–antioxidation” coordinated regulatory network and “neuro–metabolic” coupling mechanism provide a new perspective for a deep understanding of the complex regulatory mechanisms of the formation of fleshy traits (Figure 8).

## 5. Conclusions

This study systematically analyzes the molecular mechanisms that regulate intramuscular fat deposition and meat tenderness by integrating multi-omic data from chromatin accessibility, transcriptome, and proteome. Key transcription factors such as MEF2 family (MEF2A/C/D), PR, GR, AR-halfsite, NF1-halfsiteR, as well as muscle fiber type regulatory factors such as MYOG, MYF5, TCF21, AP4, and SIX2 were identified. Multi-omics joint analysis determined the core role of the metabolic genes related to *PDK4*, *LIPE*, *GPX1,* and *ART3*, as well as the extracellular matrix genes of *COL3A1*, *COL14A1,* and *VCAN*, while *DCLK1* and *MBP* act as potential regulators. Pathway analysis revealed the importance of signaling pathways such as AMPK, Apelin, HIF-1, and Arachidonic acid metabolism, ECM-receptor interaction in flesh formation. These findings not only construct a regulatory network for pork fat deposition and meat tenderness but also provide new targets and a theoretical basis for molecular breeding.

## Data Availability

None of the data was deposited in an official repository. Data will be made available on request.

## Supplementary Materials

The following supporting information can be downloaded at: https://www.mdpi.com/article/10.3390/biom15121738/s1, Table S1: qPCR primer sequence; Table S2: Phenotypic Data; Table S3: ATAC-seq quality control; Table S4: differentially accessible genes; Table S5: differential open chromatin region; Table S6: GO Analysis of DAGs; Table S7: KEGG Analysis of DAGs; Table S8: RNA-seq quality control; Table S9: GO Analysis of DEGs; Table S10: KEGG Analysis of DEGs; Table S11: GO Analysis of DEPs; Table S12: KEGG Analysis of DEPs.
